# Use of Equivalent Loss Models Under Section 316(b) of the Clean Water Act

**DOI:** 10.1100/tsw.2002.224

**Published:** 2002-06-13

**Authors:** William Dey

**Affiliations:** ASA Analysis & Communication, Inc., 291 County Route 62, New Hampton, NY 10958, USA

**Keywords:** impact assessment, population modeling, cooling water intakes, 316(b), fish

## Abstract

Equivalent loss models encompass a variety of life table-based approaches that can be used to convert age- and life stage-specific estimates of entrainment and impingement loss to a common, easily understood currency. This common currency can be expressed in terms of numbers of individuals, yield to the fishery, or biomass to the ecosystem. These models have at least two key uses in the Section 316(b) assessment process: screening for adverse environmental impact (AEI) and determination of environmental benefits associated with intake alternatives. This paper reviews the various forms of equivalent loss models, their data input requirements, and their assumptions and limitations. In addition, it describes how these models can be used as a second-level screening tool as part of the assessment of the potential for AEI. Given their relative simplicity and ease of use, equivalent loss models should prove to be an important tool in the arsenal of impact assessment methods for Section 316(b).

### INTRODUCTION

Section 316(b) of the Clean Water Act requires that a cooling-water intake reflect the best technology available (BTA) to minimize adverse environmental impact (AEI). This section of the Act has traditionally been addressed in two steps. First, there is the issue of whether or not the intake as proposed or constructed will result or has resulted in an AEI. Although there is currently no clear regulatory guidance as to what constitutes an AEI, assessments have most commonly focused on effects to populations of aquatic organisms inhabiting the source water body for the cooling water[1]. Such population level effects can result from the loss of organisms through one of two processes: *entrainment,* the passage of smaller, typically planktonic organisms through the cooling system along with the cooling-water flow, and *impingement,* the entrapment of larger aquatic organisms against the intake screens. Both of these processes can result in the mortality of organisms.

The second step in the 316(b) determination process is selection of the BTA to minimize any AEIs expected to occur. As with the concept of AEI, little regulatory guidance exists for the selection of the BTA. However, based on case law and practice, "best technology" has been typically interpreted to mean a proven intake technology that could be installed at a cost not wholly disproportionate to the environmental benefits.

Both steps in the 316(b) determination process require biological information about the aquatic populations in the source water body. Over the years, a variety of modeling approaches have been used in each step of the determination process. One approach has been to use a class of models to estimate the equivalent losses resulting from entrainment and impingement. While specific variations of these models have been used for 316(b) determinations for many years, these techniques have been recently expanded to make them even more relevant for both impact assessment and estimation of the environmental benefits of installing cooling-water intake structures. The purpose of this paper is to provide a brief overview of this class of models, to discuss their strengths and weaknesses, to provide some guidance for selection of input parameters, and to provide recommendations as to their most appropriate incorporation into the determination process under 316(b).

### BACKGROUND

Use of equivalent loss models for the assessment of power plant impacts was first suggested by Horst in his review of methods for assessing impacts of entrainment of ichthyoplankton[2]. Horst's proposed method was described as a "simplistic approach … to translate the number of ichthyoplankters lost to entrainment into the number of equivalent adults that would have resulted assuming no compensatory mechanisms in the population." If we assume a population in equilibrium, then total fecundity produced by a breeding pair over their lifetime would result in the average survival of two breeding adults to the next generation. In other words, the lifetime fecundity of a single female is expected to result in the replacement of that female and a mate if the population is to neither increase nor decrease. Under such a scenario, Horst reasoned that overall average survival across a generation could be estimated as follows:

$$S_{e \to a} = \frac{2}{FEC_L} \tag{1}$$

where $S_{e \to a}$ is the overall survival from egg to adult, 2 is the average number of surviving adults, and $FEC_L$ is the lifetime fecundity of a breeding pair.

Consequently, if the entrained organisms are all eggs, then the number of equivalent adults (NA) expected to result from the entrained eggs can be defined as:

$$NA = NE_{eggs} \times S_{e \to a} \tag{2}$$

where $NE_{eggs}$ is the number of eggs lost to entrainment.

Horst further reasoned that if $S_{e \to a}$ is the survival from egg to larval stage and $S_{l \to a}$ is survival from larval to adult, then

$$S_{e \to a} = S_{e \to l} \times S_{l \to a} \tag{3}$$

and

$$S_{l \to a} = \frac{S_{e \to a}}{S_{e \to l}} = \frac{2}{F \times S_{e \to l}} \tag{4}$$

Thus, if the entrained organisms are larvae instead of eggs, the number of equivalent adults becomes:

$$NA = NE_{larvae} \times S_{l \to a} \tag{5}$$

where $NE_{larvae}$ is the number of larvae lost to entrainment.

Horst concluded that the resulting number of equivalent adults could be compared to some reference, such as catch statistics for commercial or sport species, as part of a population-level impact assessment.

Subsequently, Goodyear expanded on Horst's model to include multiple life ages or stages entrained as follows[3]:

$$NA = \sum_{i=1}^{n_e} \left( NE_i \times S_{i \to a} \right) \tag{6}$$

where $NA$ is the total number of equivalent adults, $NE_i$ is the number of life stage or age (i) entrained, $S_{i \to a}$ is the survival from life stage or age (i) to adult, and $n_e$ is the total number of life stages or ages entrained.

Further, Goodyear identified that ages or life stages for this analysis could be defined on either an age or length basis. He also established that the lifetime fecundity used to estimate total eggs-to-adult survival ($FEC_L$) should be based on the expected lifetime fecundity of a female entering the adult population, as follows:

$$FEC_L = \sum_{J=a}^{m} \left( FM_j \times S_{r \to j} \times FEC_j \right) \tag{7}$$

where $FM_j$ is the fraction of females that are mature in age class ($j$), $S_{r \to j}$ is the survival from recruitment to adult age class (j), $FEC_j$ is the average fecundity of mature female of age class ($j$), $a$ is the age at recruitment to adult, and $m$ stands for the oldest age classes in the population.

Finally, Goodyear stated that the equivalent number of fish lost to the fishery (NF) could be estimated from the number of equivalent adults (NA) as follows:

$$NF = \frac{(NA \times F_a)}{Z_a} \tag{8}$$

where $Z_a$ is the instantaneous total mortality rate for adults and $F_a$ is the instantaneous fishing mortality rate for adults.

Horst's and Goodyear's model, commonly referred to as the Equivalent Adult Model (EAM), has been widely adopted as part of the suite of techniques used to assess the potential for AEI of cooling-water withdrawals[4].

Subsequent to these two seminal publications, impact assessors realized that the EAM approach was equally useful for assessment of potential effects of impingement as well as entrainment. In addition, it was determined that the EAM framework could be used to estimate the equivalent loss of individuals at any selected life stage, not just adults. For example, the EAM framework could be used to estimate the equivalent loss in reproductive effort (e.g., eggs) resulting from entrainment or impingement of older life stages[5]. Further, this approach could be used to estimate the number of individuals at a specific life stage (e.g., juveniles or fingerlings) that could be replaced through stocking or habitat improvements[6]. However, it is important to recognize that the number of equivalent individuals is dependent on the age endpoint selected for the calculation. For example, the loss of 1 million larvae might be equivalent to the loss of 100 individuals at age 1 but only 1 individual at age 5. Thus, it is important that the age of equivalency selected be most relevant to the impact assessment goals.

Further, assessors recognized that this same framework could be extended to address two additional assessment endpoints beyond the equivalent number of adults: equivalent yield to the fishery and equivalent amount of forage lost. Equivalent yield to the fishery allows estimation of total yield (in weight) that could have accrued to a commercial or recreational fishery from those individuals lost to entrainment or impingement in the absence of compensatory changes in total mortality. Calculation of equivalent yield integrates Baranov's catch equation[7], similar to the concept of the equivalent number of fish lost to the fishery as defined by Goodyear, with estimates of the mean weight by age. This equivalent yield is estimated as follows:

$$EY = \sum_{i=1}^{n_f} \left[ \frac{NA_i \times V_i \times F_i \times A_i}{Z_i} \times W_i \right] \quad (9)$$

where $EY$ is the equivalent yield to the fishery, $NA_i$ is the equivalent number at the beginning of each age estimated using the EAM sequentially for each Age (*i*), $V_i$ is the vulnerability of Age (*i*) to fishing, $F_i$ is the instantaneous fishing mortality rate for Age (*i*), $Z_i$ is the instantaneous total mortality rate for Age (*i*), $A_i$ is the total mortality rate for Age (*i*) (equal to $1\text{-}e^{-Zi}$), $W_i$ is the average weight for individual of Age (*i*), and $n_f$ is the maximum number of Ages (*i*) vulnerable to fishery.

This method, the Equivalent Yield Model (EYM), results in an estimate of yield defined in the same units used to describe the average weight of the individuals (e.g., lb or kg) and integrates yield across the entire lifetime of surviving individuals. This method is clearly most relevant for species with active commercial or recreational fisheries. As with the EAM, the results assume no compensatory changes in natural mortality rates. This model has been used to address the effects of entrainment and impingement at several power plants[8,9].

For aquatic organisms whose principal ecological role is to serve as food for larger predators (e.g., minnows, anchovies) or otherwise provide energy for other trophic levels, the number of individuals lost expressed as the number of adults is a measure of little direct relevance to man. Further, without any commercial or recreational harvest, the potential yield to a fishery is also not relevant. For these species, then, what is important is the amount of biomass that could be used as energy for other trophic levels, including many predators that are directly harvested by man.

For these species, it is the cumulative mortality of the population across all life stages and ages that provides the biomass for other trophic levels, assuming this mortality is largely a result of predation. Thus, for such species, a useful and relevant measurement endpoint is the total cumulative biomass, which otherwise would have been consumed by other trophic levels, that was lost to the system as a result of entrainment and impingement at cooling-water intakes. Using a framework similar to both EAM and EYM, it is then possible to estimate the mortality occurring in each life stage and multiply the result by the average weight of each life stage to determine the total amount of biomass that would have resulted from the subsequent consumption of the individuals had entrainment or impingement not occurred. This equivalent biomass lost is calculated as follows:

$$BL = \sum_{i=1}^{n_L} \left\{ \sum_{j=1}^{n_e} \left[ N_j \times \left( S_{j,i} - S_{j,i+1} \right) \times W_i \right] \right\} \tag{10}$$

where $BL$ is the equivalent biomass lost, $N_j$ is the number of life stage or age ($j$) lost to entrainment or impingement, $S_{j\rightarrow i}$ is the cumulative survival from life stage or age ($j$) to beginning of age ($i$), $S_{j\rightarrow i+1}$ is the cumulative survival from life stage or age ($j$) to beginning of age ($i$ +1), $W_i$ is the average weight of life stage ($i$), $n_e$ is the total number of life stages or ages ($j$) entrained or impinged, and $n_L$ is the total number of life stages or ages ($i$) up to maximum life span.

This method, the Biomass Lost Model (BLM), results in an estimate of biomass lost defined in the same units used to describe the average weight of the individuals and integrates this loss across all ages. While this method is specifically designed to address the loss of forage species, the BLM can also be applied to the earlier life stage of commercial and recreational species when natural mortality rates (presumably as a result of predation) are high. As with both the EAM and the EYM, the results assume no compensatory changes in natural mortality rates. The BLM is conceptually similar to the Production Foregone Model proposed by Rago[10] and Jensen[11]. The BLM has been used to estimate the effects of entrainment and impingement at several power plants[9,12,13,14,15].

As a result of these advances, there now exist three variations of equivalent loss models — the EAM, the EYM, and the BLM — all of which are based on the approach originally proposed by Horst[2] and Goodyear[3]. These three models address different measurement endpoints that result from the three possible fates that can befall an individual passing through a life stage: (1) surviving to next stage, (2) being caught by a fisherman, or (3) being consumed by other trophic levels. Each of these endpoints can have relevance to the assessment of AEI and to the determination of ecological benefits of potential alternative intake technologies. Each model can be implemented in spreadsheet software with minimal programming expertise.

## SELECTION OF MODEL INPUTS

All three versions of equivalent loss models require three common life stage/age–specific input parameters: estimates of entrainment and impingement loss, estimates of rate of mortality for each life stage/age in the population, and estimates of the duration of each life stage/age. In addition, the EYM and BLM both require estimates of life stage/age–specific average weights and the EYM requires estimates of age-specific fishing vulnerability and mortality rates. Each of these input parameters is described below.

### Entrainment and Impingement Loss Estimates

Estimates of entrainment and impingement loss are most commonly made on an annual basis and are generated for each vulnerable life stage of each species that is the target of the assessment. Typically, these estimates of loss are based on site-specific sampling that is scaled up to the total flow of the intake and adjusted for collection efficiency, potential recirculation, and entrainment/impingement mortality. The general form of this calculation is as follows:

$$NL_i = \sum_{s=1}^{q} \left[ \frac{D_{si} \times CW_s}{CE_{si}} \times PM_{si} \right] \qquad (11)$$

where $NL_i$ is the estimated number life stage/age ($i$) lost to entrainment or impingement, $D_{si}$ is the density life stage/age class ($i$) entrained or impinged during sampling period ($s$), $CE_{si}$ is the collection efficiency of life stage/age class ($i$) collected during sampling period (s), $PM_{si}$ is the entrainment or impingement mortality at the plant for life stage/age class ($i$) during sampling period ($s$), $CW_s$ is the total cooling water flow for the plant during sampling period ($s$), and $q$ is the total number of sampling periods ($s$) in the estimation interval (typically 1 year).

Details on collecting site-specific entrainment and impingement data and the subsequent estimation of losses are not discussed further as they are highly site specific.

### Population Mortality Rates

*Mortality rates* refer to the probability of death of an individual. Mortality rates are often expressed as instantaneous rates[7] and the total instantaneous mortality rate combines the effects of mortality from fishing and from all other sources (lumped under *natural mortality*) such that:

$$Z_i = F_i + M_i \qquad (12)$$

where $Z_i$ is the instantaneous total mortality rate for life stage/age($i$), $F_i$ is the instantaneous fishing mortality rate for life stage/age($i$), and $M_i$ is the instantaneous natural mortality rate for life stage/age($i$).

Obviously, for species and/or life stages that are not fished, then $F_i = 0$, and the total mortality rate equals the natural mortality rate (i.e., $Z_i = M_i$).

The complement of mortality is survival, such that:

$$S_i = e^{-(Z_i \times t_i)} \qquad (13)$$

where $S_i$ is survival during life stage/age ($i$) and $t_i$ is the duration of life stage/age ($i$).

Estimates of life stage– and age-specific mortality rates, particularly for the older ages, are often available from the scientific literature. This is especially true for species of commercial and/or recreational importance, where there is an increasing desire to manage these species through the use of quantitative models that require much of the same information as required by the models described in this paper.

However, it is often the case that reliable population mortality rates are not available for all life stages and ages. Thus, it is up to the assessor to select the most appropriate mortality rates for equivalent loss estimation. One commonly used tool for this selection process is a *life table.* A life table is a technique used to track life stage– and age-specific population parameters, such as mortality, maturity, sex ratios, and fecundity[16]. Also displayed in a life table is the integration of all parameters in their effects on subsequent population behavior.

One common simplifying assumption for selection of life stage– and age-specific mortality rates is that the population is at equilibrium — that is, that the population is neither increasing nor decreasing. This is the assumption used by Horst[2] and Goodyear[3] in their development of the EAM. Yet it is clear that populations are rarely, if ever, at equilibrium, particularly when considered on a short-term basis. Instead, they fluctuate to higher and lower levels of abundance as a result of a variety of abiotic and biotic factors. However, assuming the population is neither going extinct nor increasing to significantly higher levels, most populations tend to fluctuate around some long-term average[16]. It is this long-term average that represents equilibrium conditions. Thus, use of an equilibrium assumption appears appropriate for determining the long-term effects of entrainment and impingement over the life of a power plant (typically 20 to 30 years or more).

As noted above, under equilibrium conditions the total survival *(S)* across a generation is fixed at:

$$S_{e \to a} = \frac{2}{FEC_L} \tag{14}$$

This occurs when the expected survival of a female egg is 1 (i.e., when a female just replaces herself each generation):

$$1 = \sum_{i=1}^{n_L} \left( S_{e \to i} \times PF_i \times FM_i \times FEC_i \right) \tag{15}$$

where $S_{e \to i}$ is the cumulative survival from egg to life stage/age (*i*), $FM_i$ is the fraction of life stage/age (*i*) females that are mature, $PF_i$ is the proportion of life stage/age (*i*) that are female, and $F_i$ is the mean fecundity of life stage/age (*i*).

Using this relation, it is possible to adjust the mortality rates within the life table so that the cumulative survival is *S* and the population comes into equilibrium. There are a variety of techniques that could be used for this adjustment process. For example, it is likely that the assessor will have greater confidence in some of the estimates of mortality than others. In fact, it is common that estimates for some life stages and ages might be missing altogether. One approach, then, would be to fix the estimates with the highest degree of certainty and iteratively vary the others until arriving at internally consistent and biologically meaningful estimates of mortality. Another approach would be to assume some underlying functional relationship between natural mortality and a known biological measure such as size[17,18]. This functional relationship could then be used as guidance to adjust the available estimates of life stage–specific mortality to generate the inputs needed for equivalent losses estimation.

### STAGE/AGE DURATIONS

Typically, the older ages are defined on an annual basis (e.g., age 1, age 2, etc.). For these ages, durations are fixed at 1 year (i.e., 365 days). Younger individuals are commonly categorized by developmental stage (e.g., egg, yolk-sac larvae, post yolk-sac larvae, etc.). The durations of these stages are dependent on the development rate of the individual, and hence are typically a function of water temperature. For the purposes of equivalent loss modeling, average stage durations are commonly used, although it is possible to have variable life stage durations as well. Finally, it is possible to assign the early life stages of fish to specific ages (e.g., days) through the use of microstructure analysis of otoliths[19]. While this approach could reduce the uncertainty resulting from variable state durations, such a practice is not common owing to the labor-intensive requirements of the otolith analysis.

It is important to determine the age of the individuals lost to entrainment or impingement in addition to the total duration of each life stage/age. This is especially important for the larval stages with high natural mortality rates. For example, substantially different equivalent loss estimates could result depending on whether entrained post yolk-sac larvae came from the beginning, middle, or end of the total duration of this life stage. Typically, three approaches have been used to estimate the specific age of individuals within a life stage/age category. First, use of otolith analysis can provide actual ages of fish. However, as previously noted, this practice is not common because of high labor requirements. Second, analysis of length-frequency distributions within individual life stages/ages can provide insight as to whether the individuals came from early or late within the stage/age category. Finally, one could assume that all individuals within a stage/age category are equally vulnerable. In that case, the age could be assigned to the median age of surviving individuals within the category. This median age is a function of the mortality rate within the category and is calculated as follows[8]:

$$ma_i = \frac{\ln 2 - \ln(1 + e^{-Z_i t_i})}{Z_i} \tag{16}$$

where $ma_i$ is the median age of life stage/age ($i$), $t_i$ is the duration of life stage/age ($i$), and $Z_i$ is the instantaneous mortality rate for life stage/age ($i$).

Regardless of the method used to estimate the age of individuals with a life stage/age, for the purposes of estimating equivalent loss, individuals entrained or impinged are assumed to be exposed to this mortality only from the estimated age of the individuals through the end of that life stage/age (i.e., $t_i - ma_i$).

### Average Weights

Both the EYM and the BLM require estimates of life stage/age–specific average weights. However, the weight requirements of each model are slightly different conceptually. The EYM requires average weights of individuals harvested by the fishery, whereas the BLM requires average weights of those consumed as prey. Depending on the nature of the fishery and of predation, these weights could be slightly different for the same life stage/age. For example, principal harvests for many anadromous fish species occur during spawning runs. In that case, the weights for the EYM would be heavily weighted towards individuals in the early part of the annual growth cycle. On the other hand, these same individuals are likely to be equally vulnerable to predation throughout the year with an average weight equal to the median weight of individuals passing through that life stage/age.

Information on average weights for life stages or ages is readily available for many species from the scientific literature. Alternatively, weights can be derived by combining known life stage/age–specific lengths and length-weight relationships to calculate the geometric mean of the average weight at the beginning and end of the interval. For time-specific fisheries, average life stage/age–specific weights can be estimated from fishery monitoring studies.

#### Fishing Vulnerability and Mortality Rates

Estimates of life stage/age–specific fishing and vulnerability rates are needed for the EYM. Since this model only applies to species that are actively harvested, estimates of these two rates can often be obtained from fishery management plans or from local resource management agencies. For species with specific size limits, vulnerability can often be estimated from age-specific growth rates or length frequency distributions.

### EXAMPLES OF USE

This section presents three examples of how equivalent loss models might be used as part of the overall 316(b) determination process. All examples are hypothetical and do not reflect data from any specific power plants. Population input parameters (e.g., mortality rates) were selected to reflect possible values for each species. However, the author makes no warranty as to their accuracy. Each is designed to illustrate one of the three measurement endpoints of equivalent loss: equivalent adults, equivalent yield to fishery, and biomass lost. Each will also show how this information might be used in the 316(b) process.

#### Equivalent Adults

A power plant withdraws its cooling water from nearshore marine waters along the Southern California Bight. This area is inhabited by a typical complement of nearshore marine fish species, including queenfish, a small member of the drum family. Queenfish are a popular target of pier fishermen along the coast of Southern California. Entrainment and impingement sampling at the power plant results in annual estimates of queenfish lost as follows:

| Life stage | Number lost |
|---|---|
| Eggs | 300,000,000 |
| Yolk-sac larvae | 100,000,000 |
| Post yolk-sac larvae | 50,000,000 |
| Age 0 | 24,000 |
| Age 1 | 10,000 |

Queenfish typically mature at the end of their second year of life and this age was used to define the adult stage for purposes of equivalent adult estimation.

Based on the EAM, the estimates of entrainment and impingement loss at this hypothetical power plant are equivalent to slightly more than 68,000 adults (Table 1). The regulatory agency was concerned that this level of could not be easily dismissed and required additional assessment at a population level before they could make a determination as to the potential for AEI for queenfish at this power plant.

### Equivalent Yield

A power plant withdraws its cooling water from a large (1,000-acre) lake inhabited by a typical complement of warm-water fish species, including bluegill sunfish. Entrainment and impingement sampling at the power plant results in annual estimates of bluegills lost as follows:

| Life stage | Number lost |
|---|---|
| Eggs | 0 |
| Yolk-sac larvae | 0 |
| Post yolk-sac larvae | 1,500,000 |
| Young of year | 2,500 |
| Age 1 | 400 |

In this lake, bluegills are a popular target of recreational fishermen and there was concern that the cooling-water withdrawals would reduce yield to the fishermen. Bluegills are presumed to enter the fishery when they are 6 in. long and 4 years old; the current annual instantaneous fishing mortality rate (F) is assumed to be 0.2.

**TABLE 1**
**Example of the EAM for Queenfish Based on Entrainment and Impingement Losses at a Hypothetical Power Plant**

| Life Stage/Age | Duration in days[1] | Instantaneous Mortality Rate[1] | Life Stage Total Survival Rate[2] | Cumulative Survival to Adult[3] | Number Lost to Cooling-Water Withdrawals[1] | Equivalent Adults[4] |
|---|---|---|---|---|---|---|
| Eggs | 2 | 0.250000 | 0.606530660 | 0.000014326 | 300,000,000 | 4,298 |
| YSL | 16 | 0.217432 | 0.030840963 | 0.000036811 | 100,000,000 | 3,681 |
| PYSL | 28 | 0.119659 | 0.035068962 | 0.001188686 | 50,000,000 | 59,434 |
| Age 0 | 335 | 0.010000 | 0.035084354 | 0.033895164 | 24,000 | 813 |
| Age 1 | 365 | 0.001899 | 0.500000000 | 0.666666667 | 10,000 | 6,667 |
| | | | | | **Totals** | **68,227** |

[1] Model inputs.
[2] Total survival across life stage = Exp($-Z_i * t_i$).
[3] Calculated from median age ($d_i$) to adult.
[4] Number lost times cumulative survival to adult.

Estimates of equivalent yield, using a combination of site-specific data and information from similar water bodies, produced estimates of equivalent yield of 53 kg/year (Table 2). The permitting authority then compared this estimate of lost yield, equal to less than 0.05 kg/acre, to the current annual recreational harvest (0.5 kg/acre). Based on this comparison, the authority concluded that entrainment and impingement losses were not likely to result in an AEI as such losses were a tiny fraction (approximately 10%) of the sustained annual harvest by recreational fishermen.

### Biomass Lost

A power plant withdraws its cooling water from the mesohaline section of an estuary located along the mid-Atlantic coast. Within this estuary, bay anchovy is an important prey for a variety of predatory fish, many of which support valuable commercial or recreational fisheries. Entrainment and impingement sampling at the power plant results in annual estimates of bay anchovies lost as follows:

| Life stage | Number lost |
|---|---|
| Eggs | 600,000,000 |
| Yolk-sac larvae | 300,000,000 |
| Post yolk-sac larvae | 750,000,000 |
| Age 0 | 8,000,000 |
| Age 1 | 250,000 |
| Age 2 | 50,000 |

### TABLE 2
## Example of the EYM for Bluegill Based on Entrainment and Impingement Losses at a Hypothetical Power Plant

| Life Stage/ Age | Duration in days[1] | Instantaneous Mortality[1] *Natural* | Instantaneous Mortality[1] *Fishing* | Fishing Vulnerability Rate[1] | Numbers Lost to Cooling-Water Withdrawal[1] | Equivalent Number Entering Stage/Age: Nonvulnerable[2] | Equivalent Number Entering Stage/Age: Vulnerable[3] | Equivalent Number Entering Stage/Age: Total | Equiva-lent Catch[4] | Weight per Fish (g)[1] | Equiva-lent Yield (kg)[5] |
|---|---|---|---|---|---|---|---|---|---|---|---|
| Eggs | 2.5 | 0.250000 | 0.000000 | 0.0000 | — | — | — | — | — | 0.1 | — |
| YSL | 5.5 | 0.250000 | 0.000000 | 0.0000 | — | — | — | — | — | 0.1 | — |
| PYSL | 22 | 0.250000 | 0.000000 | 0.0000 | 1,500,000 | — | — | — | — | 0.5 | — |
| Age 0 | 335 | 0.006560 | 0.000000 | 0.0000 | 2,500 | 12,210 | — | 12,210 | — | 3.0 | — |
| Age 1 | 365 | 0.001096 | 0.000000 | 0.0000 | 400 | 1,856 | — | 1,856 | — | 12.0 | — |
| Age 2 | 365 | 0.001096 | 0.000000 | 0.0000 | — | 1,565 | — | 1,565 | — | 37.0 | — |
| Age 3 | 365 | 0.001096 | 0.000000 | 0.0000 | — | 1,049 | — | 1,049 | — | 73.0 | — |
| Age 4 | 365 | 0.001096 | 0.000500 | 1.0000 | — | — | 703 | 703 | 97 | 116.0 | 11.3 |
| Age 5 | 365 | 0.001096 | 0.000500 | 1.0000 | — | — | 471 | 471 | 65 | 159.0 | 10.4 |
| Age 6 | 365 | 0.001096 | 0.000500 | 1.0000 | — | — | 316 | 316 | 44 | 200.0 | 8.7 |
| Age 7 | 365 | 0.001096 | 0.000500 | 1.0000 | — | — | 212 | 212 | 29 | 238.0 | 7.0 |
| Age 8 | 365 | 0.001096 | 0.000500 | 1.0000 | — | — | 142 | 142 | 20 | 272.0 | 5.3 |
| Age 9 | 365 | 0.001096 | 0.000500 | 1.0000 | — | — | 95 | 95 | 13 | 301.0 | 4.0 |
| Age 10 | 365 | 0.001096 | 0.000500 | 1.0000 | — | — | 64 | 64 | 9 | 325.0 | 2.9 |
| Age 11 | 365 | 0.001096 | 0.000500 | 1.0000 | — | — | 43 | 43 | 6 | 345.0 | 2.0 |
| Age 12 | 365 | 0.001096 | 0.000500 | 1.0000 | — | — | 29 | 29 | 4 | 365.0 | 1.4 |
| | | | | | | | | | | Total | 53.0 |

1 Model inputs.
2 Equivalent number surviving not vulnerable to fishing assuming that actual loss is median age for life stage ($d_i$).
3 Equivalent number surviving vulnerable to fishing assuming actual loss is median age for life stage ($d_i$).
4 Expected number harvested based on number vulnerable and Baranov's catch equation.
5 Expected number harvested multiplied by average weight.

### TABLE 3
## Example of the BLM for Bay Anchovy Based n Entrainment and Impingement Losses at a Hypothetical Power Plant

| Life Stage/ Age | Dura-tion in days[1] | Instantan-eous Total Mortality Rate[1] | Life Stage Total Survival Rate[2] | Eggs | YSL | PYSL | Age 0 | Age 1 | Age 2 | Number Consumed[4] | Average Weight (gm)[1] | Biomass Lost (kg)[5] |
|---|---|---|---|---|---|---|---|---|---|---|---|---|
| Number Lost to Entrainment/Impingement[1] | | | | 60,000,000 | 30,000,000 | 75,000,000 | 800,000 | 25,000 | 5,000 | | | |
| | | | | Number That Would Have Survived to Each Age[3] | | | | | | | | |
| Eggs | 1 | 1.044000 | 0.3520437 | | | | | | | 28,754,529 | 0.0010 | 28.8 |
| YSL | 2 | 0.78300 | 0.2088790 | 31,245,471 | | | | | | 44,351,705 | 0.0010 | 44.4 |
| PYSL | 32 | 0.19140 | 0.0021879 | 6,526,524 | 10,367,242 | | | | | 91,529,331 | 0.0040 | 366.1 |
| Age 0 | 330 | 0.00445 | 0.2302706 | 14,280 | 22,683 | 327,473 | | | | 781,043 | 0.1130 | 88.3 |
| Age 1 | 365 | 0.00445 | 0.1970594 | 3,288 | 5,223 | 75,407 | 299,473 | | | 324,610 | 1.5730 | 510.6 |
| Age 2 | 365 | 0.00445 | 0.1970594 | 648 | 1,029 | 14,860 | 59,014 | 8,231 | | 88,782 | 3.9270 | 348.6 |
| | | | | | | | | | | | Total | 1,386.7 |

1 Model inputs.
2 Total survival across life stage = $Exp(-Z_i * t_i)$.
3 Equivalent number surviving to each age assuming actual loss is median age for life stage ($d_i$).
4 Assuming all dead are consumed.
5 Number consumed times average weight.

The permitting authority wanted to know if an alternative to the existing intake, which could reduce these losses but would cost an average of $1.5 million/year, could be justified from an economic perspective.

Using a combination of site-specific data and information from similar water bodies, the BLM produced estimates of biomass lost from entrainment and impingement of approximately 1,387 kg/year (Table 3). Since the permitting authority wanted to make an economic comparison and direct economic values of bay anchovy are uncertain, this biomass lost was converted to an equivalent weight of recreational and commercial species for which economic values are available. Assuming a 10% conversion efficiency, this biomass lost equates to a biomass of 1,387 kg of commercial or recreational fish, assuming that all bay anchovies would have been consumed by these species. Using an average value of $20/kg of commercial or recreational fish, the biomass of bay anchovy lost would have an economic value of $27,800/year expressed as commercial or recreational fish.

Presuming the intake alternative has the potential to reduce these losses by 80%, then the economic benefit of this alternative to bay anchovy would be $22,240/year. However, by comparing this expected annual benefit to the estimated annual cost for the intake alternative ($1.5 million), the permitting authority concluded that the intake alternative could not be justified on a cost-benefit basis.

### CONCLUSIONS AND RECOMMENDATIONS

The class of models discussed in this paper (equivalent loss models) can be a useful and relatively simple tool for making determinations under 316(b) for several reasons.

First, this approach provides loss measures in common currency (numbers, fishery yield, or biomass) that can be used to address the question of AEI. For a relatively few high-profile cases in which population-level losses are of concern, these models are unlikely to be sufficient for final determination. In these cases, more complex population-based assessment techniques will be required (as we saw in the Equivalent Adult hypothetical example). However, for many cooling-water intakes, losses are relatively small and the use of equivalent loss models can be all that is required to demonstrate that these losses are relatively small compared to acceptable levels of harvest (as we saw in the Equivalent Yield hypothetical example).

Second, equivalent loss models provide measures of loss that are easily recognizable by the lay public (e.g., number or pounds of fish). This allows the public to better understand management decisions that are being made regarding public resources.

Third, the results of equivalent loss modeling are defined in units that can be directly translated into a consideration of cost and benefits of management decisions (as we saw in the Biomass Lost hypothetical example). While the role of explicit cost-benefit analysis in the 316(b) determination process remains to be determined, some consideration of relative costs vs. resulting environmental benefits is likely to be involved in these decision-making processes, as it is with many other environmental management questions.

Fourth, all of the models described in this paper can be easily implemented using readily available spreadsheet software. Not special knowledge of ecological modeling or computer programming is required.

It is important to recognize, however, that despite the advantages of equivalent loss modeling listed above, these approaches require biological information not readily available for many aquatic populations (e.g., life stage specific mortality rates). Uncertainty in these model inputs can be addressed through sensitivity analysis, Monte Carlo simulations, or fuzzy arithmetic[20]. In addition, I think it would be valuable for the electric utility industry and the regulatory agencies to work together to develop a mutually acceptable range for each input parameter for various categories of species. These ranges could then be combined with site-specific estimates of entrainment and impingement loss to estimate equivalent loss.
